# Autoimmune Thyroid Disease in Psoriasis Patients: A Case-Control Study

**DOI:** 10.7759/cureus.50197

**Published:** 2023-12-08

**Authors:** Praveen Prashant, Renu Garg, Usha Kataria, Sonia Vashist, Piyush Bansal, Gulshan Prakash, Sumit Dokwal, Abhishek Bansal

**Affiliations:** 1 Department of Biochemistry, Pandit Bhagwat Dayal Sharma Post Graduate Institute of Medical Sciences, Rohtak, IND; 2 Department of Biochemistry, Bhagat Phool Singh Government Medical College for Women, Sonepat, IND; 3 Department of Dermatology, Venereology, and Leprology, Bhagat Phool Singh Government Medical College for Women, Sonepat, IND; 4 Department of Dermatology, Tripti Hospital, Rohtak, IND

**Keywords:** anti-tg ab, anti-tpo ab, psoriasis and autoimmunity, aitd, subclinical hypothyroidism, hypothyroidism

## Abstract

Introduction: Psoriasis is a common immunologically mediated inflammatory disease characterized by skin inflammation, epidermal hyperplasia, an increased risk of painful and destructive arthritis, cardiovascular morbidity, and psychosocial challenges. Some autoimmune diseases are mediated by stimulating or blocking auto-antibodies. Auto-antibodies may act as antagonists and bind to hormone receptors, blocking receptor function. It may result in impaired secretion of mediators and gradual dysfunction of the affected organ, e.g., Graves disease and myasthenia gravis.

Objective: This study was planned to evaluate the association between anti-thyroid peroxidase antibody (anti-TPO Ab) and anti-thyroglobulin antibody (anti-TG Ab) as biochemical markers in 30 clinically diagnosed psoriasis patients.

Materials and methods: This hospital-based, epidemiological case-control study was conducted in the Department of Biochemistry in collaboration with the Department of Dermatology, Venereology, and Leprology at Bhagat Phool Singh Government Medical College for Women, Khanpur Kalan, Sonepat, Haryana, India. Thirty subjects diagnosed clinically with psoriasis and an equal number of age-matched controls with no known autoimmune disease from the outpatient department were also enrolled. The following hormonal tests, i.e., thyroid-stimulating hormone (TSH), free triiodothyronine (FT3), free thyroxine (FT4), and antibodies, anti-TPO Ab and anti-TG Ab, were performed. The study period was one year. The data thus obtained was analyzed using SPSS Statistics version 26.0 (IBM Corp. Released 2019. IBM SPSS Statistics for Windows, Version 26.0. Armonk, NY: IBM Corp). The significance level (p-value) was taken as <0.05.

Results: The mean age of psoriasis subjects was 37.83±12.89 years compared to 36.91±12.32 years in the control group and was found to be non-significant (p=0.432), reflecting a similar age distribution. A male preponderance was observed in the present study, where the psoriasis group consisted of 80% males and 20% females, while the control group had 60% males and 40% females. All six psoriasis patients diagnosed with autoimmune thyroid disease (AITD) were euthyroid at the time of enrollment, compared to only one control subject in a subclinical hypothyroid state. The mean values of anti-TPO Ab were 30.93±41.26 IU/mL in psoriasis patients and 11.39±28.42 IU/mL in the control group (p=0.001), while the mean values of anti-TG Ab were 11.21±27.69 IU/mL in psoriatic subjects and 2.49±9.05 IU/mL in the control group (p=0.004). No significant correlation between AITD and psoriasis was found when both parameters were analyzed statistically for correlation; even when one marker was considered, no significant correlation was found. The odds ratio was calculated to find an association between the disease and thyroid autoimmunity. The odds ratio was estimated to be 2.25 for psoriasis and the control group, with a confidence interval of 95% (0.77-6.59) and a p-value of 0.139, which was not statistically significant.

Conclusion: Psoriasis, a dermatological disorder, has been seen as related to AITD. The role of early detection of anti-thyroid antibodies, i.e., anti-TPO Ab and anti-TG Ab, can be of prognostic value in AITD and psoriasis.

## Introduction

The incidence of skin disorders is increasing exponentially in the population. The skin outpatient department encounters dermatological diseases with either direct or associated autoimmune etiologies. Skin manifestations can be a result of the underlying pathology in the body. Autoimmune disorders of the skin can be clinically diagnosed and confirmed by various biochemical markers [1].

Psoriasis is a common immunologically mediated inflammatory disease characterized by skin inflammation, epidermal hyperplasia, an increased risk of painful and destructive arthritis, cardiovascular morbidity, and psychosocial challenges. Psoriasis is universal in occurrence, and its prevalence ranges from 0.91% in the United States to 8.5% in Norway. The prevalence in Asians is though low. The age of onset of psoriasis cannot be definite, but it is uncommon before 10 years. The most typical age for the appearance of symptoms is 15-30 years. Interestingly, the prevalence of psoriasis and concordance in monozygotic and dizygotic twins decreases with decreasing distance from the equator. Such observations propound the hypothesis that UV light exposure may be a significant environmental factor. Genetic factors are also supposed to play an essential role in the etiopathogenesis of psoriasis [2].

Auto-antibodies may act as antagonists and bind to hormone receptors, blocking receptor function, resulting in impaired secretion of mediators and gradual dysfunction of the affected organ, e.g., Graves disease and myasthenia gravis. Thyroid peroxidase oxidizes iodide and acts as an H2O2 donor during the synthesis of thyroid hormones. The resulting compound may be I+ or OI-(hypoiodite), which can interact with thyroglobulin (TG). The antibody production against thyroid peroxidase (TPO), a 933 amino acid long highly glycosylated transmembrane hemoprotein of several domains, leads to thyroid dysfunction. The anti-thyroid peroxidase antibody (anti-TG Ab) was the first antibody reported to be associated with autoimmune thyroid disease (AITD). Most anti-TG Abs are of the immunoglobulin class IgG, which is more prevalent than IgA. It is an intra-follicular antibody that binds to immune cells and antigens, possibly resulting in tissue destruction in some instances. Massive destruction of the thyroid gland tissue induces structural changes in TG, leading to antibody production against TG [3-4].

Thyroid dysfunction and thyroid autoimmunity have been reported to be associated with dermatological disorders; thus, this study examined the association of biochemical markers of thyroid autoimmunity with psoriasis.

## Materials and methods

This hospital-based, epidemiological cross-sectional, case-control study was conducted in the Department of Biochemistry in collaboration with the Department of Dermatology, Venereology, and Leprology in a tertiary-care teaching hospital in Northern India, Bhagat Phool Singh Government Medical College for Women. Institutional scientific and ethical approval was obtained before the start of the study after the due procedure (approval number: BPSGMCW/RC476/IEC/19).

Selection of participants

The online epi-info© sample size calculator software (Centers for Disease Control and Prevention, Georgia, USA) was used to calculate the sample size, which came out to be 60 (30 cases and 30 controls) [5]. Thirty subjects visiting for the first time and diagnosed clinically with psoriasis and an equal number of age-matched controls with no known autoimmune disease from the outpatient department were also enrolled. Pregnant females, subjects with known thyroid disease, thyroid surgery, thyroid medication, subjects with any acute or chronic systemic illness, and any other autoimmune disease that may mimic or hamper the results were excluded from the study.

The purpose of the study in the vernacular was explained to the participants, and a patient information sheet was provided to them. After obtaining voluntary written consent, the subjects were assigned to group 1, i.e., psoriasis, and group 2, i.e., the control group.

Sample collection and processing

Subjects were instructed to fast overnight for 8-10 hours before the sample collection. A fasting venous sample was collected from all the participants following all the aseptic procedures. The following hormonal tests, i.e., thyroid-stimulating hormone (TSH), free triiodothyronine (FT3), free thyroxine (FT4), and antibodies, anti-thyroid peroxidase antibody (anti-TPO Ab) and anti-thyroglobulin antibody (anti-TG Ab), were performed in the serum on the same day using a commercially available enzyme-linked immunosorbent assay (ELISA) kit with prescribed internal quality control procedures. The normal working reference range for serum TSH was 0.35-4.25 mU/L, for serum FT3 was 2.4-4.2 pg/mL, and for serum FT4 was 0.7-1.24 ng/mL. The serum anti-TPO Ab and anti-TG Ab reference values were taken at <35 IU/mL and <40 IU/mL, respectively. An external quality assurance scheme from CMC Vellore, Tamil Nadu, India, was also sourced, and quality control was established.

We compared the characteristics of various data using the Mann-Whitney U test for quantitative data and the Chi-square test for qualitative data using SPSS Statistics version 26.0 (IBM Corp. Released 2019. IBM SPSS Statistics for Windows, Version 26.0. Armonk, NY: IBM Corp). The significance level (p-value) was taken as <0.05.

## Results

The mean age of psoriasis subjects was 37.83±12.89 years compared to 36.91±12.32 years in the control group and was found to be non-significant (p=0.432), reflecting a similar age distribution. A male preponderance was observed in the present study, where the psoriasis group consisted of 80% males and 20% females, while the control group had 60% males and 40% females (Table 1).

**Table 1:** Gender distribution in case and control groups and AITD subjects in each group AITD: autoimmune thyroid disease

Gender	Psoriasis (group 1A)/subjects with AITD	Controls (group 2)/subjects with AITD
Male	24 (80%)/4	18 (60%)/2
Female	6 (20%)/2	12 (40%)/1

A highly significant difference between the mean values of TSH, FT4, and FT3 in serum was observed in statistical analysis when the psoriasis group was compared to the controls (all p<0.001**). The mean value of anti-TPO Ab was also highly significant (p<0.001**), while anti-TG Ab levels were significantly higher in the psoriasis group than in the control group (p=0.004) (Table 2).

**Table 2:** Comparison of age and clinical variables between case and control groups Asymptotic significances (two-sided tests) are displayed. The significance level is p<0.05 (*significant), p<0.001 (**highly significant) using the Mann-Whitney U test. S. FT3: serum-free T3, S. FT4: serum-free T4, S. TSH: serum thyroid-stimulating hormone, S. anti-TPO Ab: serum anti-thyroid peroxidase antibody, S. anti-TG Ab: serum anti-thyroglobulin antibody, IU/mL: international unit per milliliter, mIU/mL: mili international unit per milliliter, ng/mL: nanograms per milliliter, pg/mL: picograms per milliliter

	Age (years)		S. FT3 (pg/mL)		S. FT4 (ng/mL)		S. TSH (mU/L)		S. anti-TPO Ab (IU/mL)		S. anti-TG Ab (IU/mL)	
N=30	Case	Control	Case	Control	Case	Control	Case	Control	Case	Control	Case	Control
Mean	37.83	35.92	2.98	3.37	0.93	1.25	1.48	3.02	30.93	11.39	11.21	2.49
Median	39.50	34.50	3.00	3.40	0.85	1.20	1.35	3.00	13.50	0.10	0.01	0.10
SD	12.89	12.32	0.35	0.46	0.25	0.33	0.89	0.93	41.26	28.42	27.69	9.05
Minimum	16	15	2.10	1.90	0.60	0.60	0.10	0.90	5.00	0.10	0.01	0.10
Maximum	59	60	3.60	5.70	1.60	2.30	4.20	8.60	159.00	144.00	135.50	67.00
p-value	0.43		<0.001**		<0.001**		<0.001**		<0.001**		0.004*	

When the prevalence of autoimmunity was compared between both groups, it was observed to be insignificant. Only six (20%) psoriasis subjects were positive for either antibody. At the same time, three of the controls were also found to suffer from AITD based on positivity for either antibody, and this difference was not significant in statistical analysis (p=0.132). One psoriasis patient and none of the control subjects were found positive for both of the antibodies (Table 3).

**Table 3:** AITD prevalence (positive for at least one antibody marker) in psoriasis and controls (odds ratio) The odds ratio, its standard error, and 95% confidence interval are calculated according to Altman (1991). The significance level is <0.05, calculated using Sheskin (2004) N: number of subjects, CI: confidence interval, Z statistics: standard score calculated

Parameter	Psoriasis N = positive subjects/30 (% age)	Controls N = positive subjects/30 (% age	p-value (Chi-square test)	Odds ratio	(CI=95%)	Z statistics
+Ve for one marker	6 (20%)	3 (10%)	0.139	2.25	0.77-6.59	1.478

All the subjects in both groups were analyzed according to individual values of serum TSH and FT4 to categorize them into euthyroid, hypothyroid, subclinical hypothyroid, and hyperthyroid status. Among subjects in both case and control groups, the maximum number of subjects diagnosed with AITD was euthyroid. In the psoriasis group, all six subjects diagnosed with AITD were euthyroid at diagnosis. In the control group, three subjects had AITD, of which two were euthyroid and one had subclinical hypothyroidism. No subject was found to have hyperthyroid status.

## Discussion

Based on the comparison of the thyroid profile, in the psoriasis group, all subjects were euthyroid, i.e., no thyroid dysfunction was observed in any psoriasis patients. Wu et al. [6] found an extremely low prevalence of 0.01% in a large sample size. The other studies demonstrated results of thyroid profile in psoriasis, which were at variance with our results [7-13].

AITD is detected most easily by measuring circulating antibodies against TPO and TG. As antibodies to TG alone are uncommon, it is reasonable to measure only anti-TPO Abs [14].

The mean values of anti-TPO Ab and anti-TG Ab were significantly higher in psoriasis subjects than in controls (p<0.001 for anti-TPO and 0.004 for anti-TG, respectively). The prevalence of AITD in the control group, i.e., healthy subjects, is found to vary between 3% and 8% in the literature [10,15,16]. The thyroid function and autoimmunity markers were assessed, and the prevalence of autoimmunity was calculated to be 1.9% by Alawneh et al. [17], while Yang et al. [14] reported it as 7.8%. Halilovic et al. [18] also reported AITD in 6% of the subjects, while Daneshpazooh et al. [19] and Sanchez et al. [20] reported 7% and 2.5% AITD in control subjects in their respective studies. A study also reported a higher prevalence of AITD than the present study in the control population.

The present study also reports an odds ratio of 2.25 with a confidence interval of 0.77-6.59 at 95%, but the association was not found to be significant when compared with the control group (p =0.139). Also, a cohort study in Taiwan reported a 0.44% prevalence of AITD [8]. It was concluded that there was a trend of increased incidence of new cases of thyroid disorder, hypothyroidism, and anti-TPO positivity, particularly in female subjects versus controls. Patients were reported to have an increased risk for the incidence of autoimmune thyroid disorders and thyroid dysfunctions; similar findings were reported in our study for the prevalence of AITD in case and control groups, but no significant association was reported in either of the studies [8,10]. Anti-TG Ab and anti-AMA Ab positivity was shown to be 5% and 14% in psoriasis subjects [15].

Psoriasis has known multi-organ involvement, and an association between thyroid dysfunction and thyroid autoimmunity in psoriatic arthropathy subjects has been suggested, though no clear-cut association between TPO Ab positivity and psoriatic disease could be established in the study [21]. In the present research, although a higher prevalence of antibodies was seen in the psoriasis group compared with the control group, a clear association was lacking. No significant relationship between thyroid autoimmunity and psoriasis was observed in a study with a prevalence of thyroid auto-antibodies reported correlating to the present study [9]. The association between psoriatic disease, Graves disease, and Hashimoto thyroiditis was not found to be significant based on an odds ratio of 1.1 (p=0.3825) and 1.2 (p=0.0822) [6]. Although anti-TPO Ab and anti-TG Ab were elevated in psoriasis and control subjects, there was no statistical significance when the two groups were compared based on thyroid auto-antibodies [13].

The present study reported a highly significant difference in anti-TPO Ab titers (p<0.001) and anti-TG Ab titers (p<0.001). The prevalence of autoimmunity based on positivity for at least one biochemical marker was calculated to be 20% (6/30). Anti-TPO Ab positivity was found in six subjects and anti-TG Ab positivity in a single subject. One-tenth of the control subjects were positive for anti-TPO Abs, but none were positive for anti-TG Abs. When compared with control subjects, the difference was not found to be statistically significant.

Some studies also found a significant prevalence and association of AITD in psoriatic patients. Thyroid dysregulation and autoimmunity were responsible factors in psoriasis [7,12]. Biochemical markers of thyroid autoimmunity, i.e., anti-TG or anti-TPO Ab positivity or thyroiditis diagnosed by ultrasonography, were significantly higher and more frequent in patients with psoriatic arthropathy [22].

The limitation of the study was the small sample size. A study with a larger sample size and a longer duration could have provided us with a better understanding of the question under study.

## Conclusions

The prevalence of AITD was almost similar in the psoriatic and control groups. However, the present study reports a statistically significant increase in the mean values of anti-thyroid antibodies and suggests an increased incidence of AITD in patients with psoriasis. The odds ratio describes the association between AITD and psoriasis, although it was not significant in our study.

## References

[1] Outerbridge CA (2013). Cutaneous manifestations of internal diseases. Vet Clin North Am Small Anim Pract.

[2] Henseler T, Christophers E (1985). Psoriasis of early and late onset: characterization of two types of psoriasis vulgaris. J Am Acad Dermatol.

[3] Williams DE, Le SN, Godlewska M, Hoke DE, Buckle AM (2018). Thyroid peroxidase as an autoantigen in Hashimoto’s disease: structure, function, and antigenicity. Horm Metab Res.

[4] Fröhlich E, Wahl R (2017). Thyroid autoimmunity: role of anti-thyroid antibodies in thyroid and extra-thyroidal diseases. Front Immunol.

[5] (2019). Sample Size: X-sectional, cohort, & randomized clinical trials. https://www.openepi.com/SampleSize/SSCohort.htm.

[6] Wu JJ, Nguyen TU, Poon KY, Herrinton LJ (2012). The association of psoriasis with autoimmune diseases. J Am Acad Dermatol.

[7] Rana A, Mahajan VK, Chauhan PS, Mehta KS, Sharma SB, Sharma A, Sharma R (2020). The association of thyroid dysfunction with chronic plaque psoriasis: a hospital-based retrospective descriptive observational study. Indian Dermatol Online J.

[8] Wang SH, Wang J, Lin YS, Tung TH, Chi CC (2019). Increased risk for incident thyroid diseases in people with psoriatic disease: a cohort study. J Am Acad Dermatol.

[9] Vassilatou E, Papadavid E, Papastamatakis P (2017). No association of psoriasis with autoimmune thyroiditis. J Eur Acad Dermatol Venereol.

[10] Fallahi P, Ferrari SM, Ruffilli I (2017). Increased incidence of autoimmune thyroid disorders in patients with psoriatic arthritis: a longitudinal follow-up study. Immunol Res.

[11] Huang KP, Mullangi S, Guo Y, Qureshi AA (2013). Autoimmune, atopic, and mental health comorbid conditions associated with alopecia areata in the United States. JAMA Dermatol.

[12] Vashist S, Mahajan VK, Mehta KS (2020). Association of psoriasis with autoimmune disorders: results of a pilot study. Indian Dermatol Online J.

[13] Gul U, Gonul M, Kaya I, Aslan E (2009). Autoimmune thyroid disorders in patients with psoriasis. Eur J Dermatol.

[14] Yang Y, Lin X, Fu W, Luo X, Kang K (2010). An approach to the correlation between vitiligo and autoimmune thyroiditis in Chinese children. Clin Exp Dermatol.

[15] Bianchi G, Marchesini G, Zoli M (1993). Thyroid involvement in chronic inflammatory rheumatological disorders. Clin Rheumatol.

[16] Rostami Mogaddam M, Iranparvar Alamdari M, Maleki N, Safavi Ardabili N, Abedkouhi S (2015). Evaluation of autoimmune thyroid disease in melasma. J Cosmet Dermatol.

[17] Alawneh S, Awad AA, Athamneh M, Abdelrahman S, Abu-Zaid M, Alnawaiseh N (2018). Association between vitiligo and thyroid autoimmunity in Jordanian population. J Immuno Biol.

[18] Kasumagic-Halilovic E, Prohic A, Begovic B, Ovcina-Kurtovic N (2011). Association between vitiligo and thyroid autoimmunity. J Thyroid Res.

[19] Daneshpazhooh M, Mostofizadeh G M, Behjati J, Akhyani M, Robati RM (2006). Anti-thyroid peroxidase antibody and vitiligo: a controlled study. BMC Dermatol.

[20] Sánchez J, Sánchez A, Munera M, Garcia E, Lopez JF, Velásquez-Lopera M, Cardona R (2021). Presence of IgE autoantibodies against eosinophil peroxidase and eosinophil cationic protein in severe chronic spontaneous urticaria and atopic dermatitis. Allergy Asthma Immunol Res.

[21] Khan SR, Bano A, Wakkee M (2017). The association of autoimmune thyroid disease (AITD) with psoriatic disease: a prospective cohort study, systematic review and meta-analysis. Eur J Endocrinol.

[22] Antonelli A, Delle Sedie A, Fallahi P (2006). High prevalence of thyroid autoimmunity and hypothyroidism in patients with psoriatic arthritis. J Rheumatol.

